# Methyl 4-(tri­fluoro­meth­yl)-1*H*-pyrrole-3-carboxyl­ate

**DOI:** 10.1107/S160053681302549X

**Published:** 2013-09-18

**Authors:** P. A. Suchetan, S. Sreenivasa, B. S. Palakshamurthy, K. E. ManojKumar, S Madan Kumar, N. K. Lokanath

**Affiliations:** a Department of Studies and Research in Chemistry, U.C.S., Tumkur University, Tumkur, Karnataka 572 103, India; b Department of Studies and Research in Chemistry, Tumkur University, Tumkur, Karnataka 572 103, India; c Department of Studies and Research in Physics, U.C.S., Tumkur University, Tumkur, Karnataka 572 103, India; d Department of Studies in Physics, University of Mysore, Manasagangotri, Mysore, India

## Abstract

In the title compound, C_7_H_6_F_3_NO_2_, all the non-H atoms except for one of the F atoms lie on a crystallographic mirror plane. In the crystal, the mol­ecules are linked into inversion dimers by pairs of C—H⋯F inter­actions, forming *R*_2_^2^(10) loops. These dimers are connected into C(6) chains along [001] through N—H⋯O hydrogen bonds. Aromatic π–π stacking inter­actions [centroid-centroid separation = 3.8416 (10) A°] connect the mol­ecules into a three-dimensional network.

## Related literature

For background to the pharmacological activity of pyrrole derivatives, see: Toja *et al.* (1987 ▶); Muchowski *et al.* (1985 ▶); Dannhardt *et al.* (2000 ▶); Burnham *et al.* (1998 ▶); Krowicki *et al.* (1988 ▶).
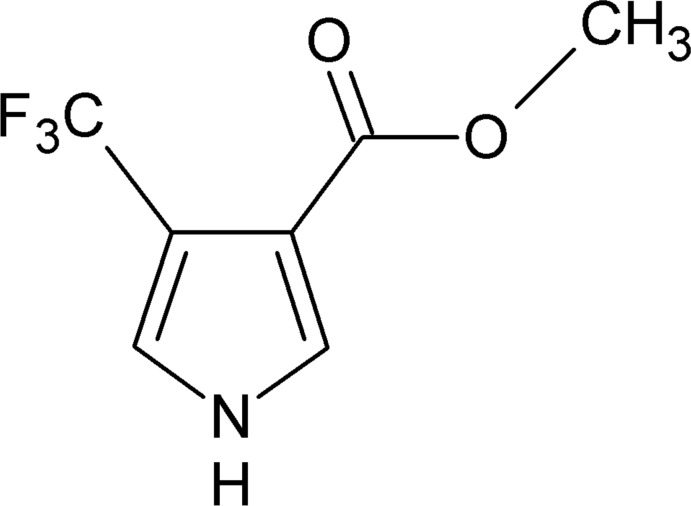


## Experimental

### 

#### Crystal data

C_7_H_6_F_3_NO_2_
*M* *_r_* = 193.13
Monoclinic, 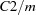

*a* = 16.643 (2) Å
*b* = 7.1118 (10) Å
*c* = 6.9618 (11) Å
β = 98.903 (7)°
*V* = 814.1 (2) Å^3^
*Z* = 4
Mo *K*α radiation
μ = 0.16 mm^−1^
*T* = 293 K
0.24 × 0.22 × 0.20 mm

#### Data collection

Bruker APEXII CCD diffractometer
Absorption correction: multi-scan (*SADABS*; Bruker, 2009 ▶) *T*_min_ = 0.963, *T*_max_ = 0.969
3752 measured reflections
707 independent reflections
645 reflections with *I* > 2σ(*I*)
*R*_int_ = 0.076

#### Refinement

*R*[*F* ^2^ > 2σ(*F* ^2^)] = 0.065
*wR*(*F* ^2^) = 0.180
*S* = 1.09
707 reflections
81 parameters
H atoms treated by a mixture of independent and constrained refinement
Δρ_max_ = 0.37 e Å^−3^
Δρ_min_ = −0.32 e Å^−3^

### 

Data collection: *APEX2* (Bruker, 2009 ▶); cell refinement: *SAINT-Plus* (Bruker, 2009 ▶); data reduction: *SAINT-Plus*; program(s) used to solve structure: *SHELXS97* (Sheldrick, 2008 ▶); program(s) used to refine structure: *SHELXL97* (Sheldrick, 2008 ▶); molecular graphics: *Mercury* (Macrae *et al.*, 2008 ▶); software used to prepare material for publication: *SHELXL97*.

## Supplementary Material

Crystal structure: contains datablock(s) I, New_Global_Publ_Block. DOI: 10.1107/S160053681302549X/hb7136sup1.cif

Structure factors: contains datablock(s) I. DOI: 10.1107/S160053681302549X/hb7136Isup2.hkl

Supplementary material file. DOI: 10.1107/S160053681302549X/hb7136Isup3.cml

Additional supplementary materials:  crystallographic information; 3D view; checkCIF report

## Figures and Tables

**Table 1:** Hydrogen-bond geometry (Å, °)

*D*—H⋯*A*	*D*—H	H⋯*A*	*D*⋯*A*	*D*—H⋯*A*
N—H⋯O1^i^	0.83 (6)	2.03 (5)	2.810 (4)	156
C5—H5⋯F1^ii^	0.93	2.52	3.442 (4)	171

Symmetry codes: (i) 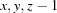; (ii) 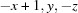.

**Fig. 1.**
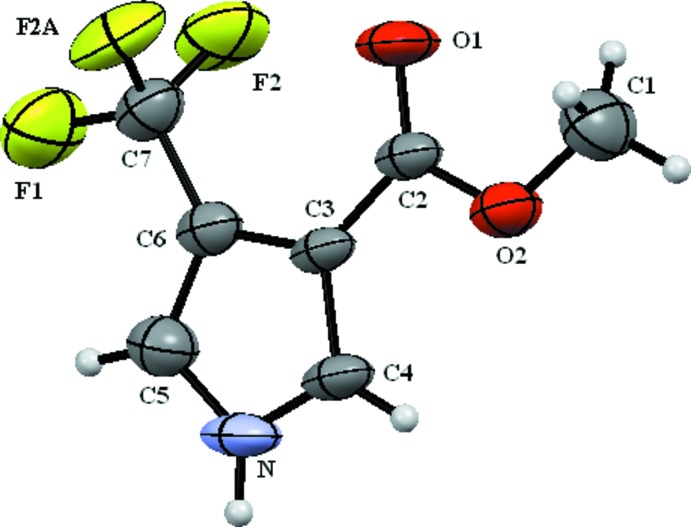
Molecular structure of the title compound, showing displacement ellipsoids drawn at the 50% probability level. Symmetry code for F2a: (x, -y, z).

**Fig. 2.**
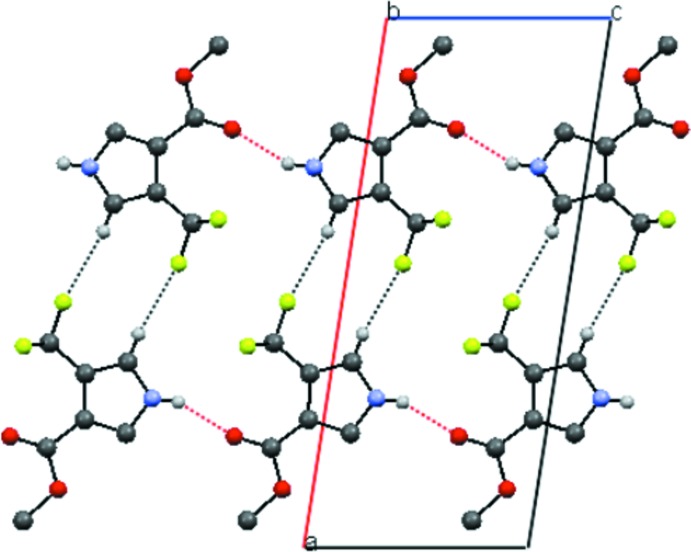
Molecular packing in the title compound displaying R22(10) loops and C(6) chains. H atoms not involved in H-bonding is omitted for clarity.

**Fig. 3.**
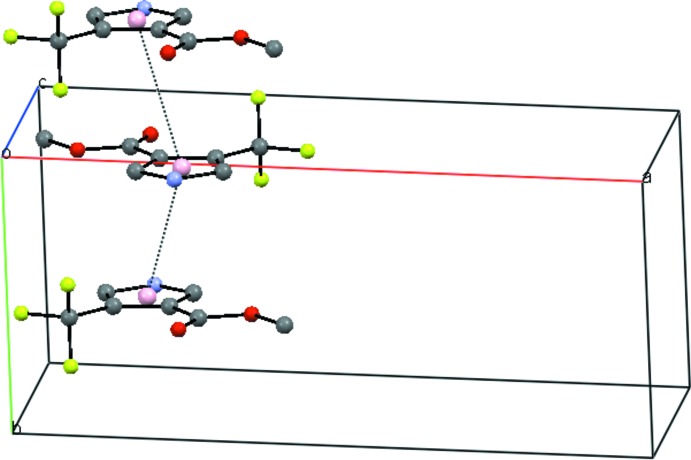
π-π stacking interactions observed in the crystal structure.

## supplementary crystallographic information

### 1. Comment

Pyrrole derivatives are given considerable attention due to their synthetic importance and their extensive use in drug discovery (Toja *et al.*, 1987) and pharmacological activity such as anti-inflammatory (Muchowski *et al*., 1985), cytotoxicity (Dannhardt *et al.*, 2000), *in vitro* cytotoxic activity against solid tumour models (Burnham *et al.*, 1998), antitumour agents (Krowicki *et al.*, 1988]) *etc*. As part of our studies in this area, the title compound was synthesized and its structure determined.

In the title compound, C_7_H_6_F_3_NO_2_, the C=O and *O*—*C*(methoxy) bonds are *syn* to each other (Fig 1). The molecules are linked into inversion dimers along *a* axis through C5—H5···F1 interactions, thus forming *R*_2_^2^(10) loops (Fig 2). These dimers are further connected into C(6) chains through strong N—H···O1 hydrogen bonds along *c* axis (Fig 2). Further, π-π stacking interactions [centroid-centroid separation = 3.8416 (10) A°] connects the molecules into a three dimensional network (Fig 3).

### 2. Experimental

Sodium hydride (0.02 mol) and methyl 4,4,4-trifluorobut-2-enoate (0.01 mol) were taken in dry Tetrahydrofuran (THF). The reaction mixture was stirred for 15 min. Toluenesulfonylmethyl isocyanide (TosMIC, 0.01 mol) was added to the reaction mixture and the mixture was heated to 50°C for 2 h. Reaction was monitored by TLC. Ethyl acetate was added to the mixture. Sodium hydride was quenched by using saturated solution of ammonium chloride and the organic layer was separated, dried and concentrated. The crude compound was purified by column chromatography using petroleum ether / ethyl acetate (7:3) as eluent to give the title compound as a colorless solid.

Colourless prisms were obtained from slow evapouration of the solution of the compound in a mixture of petroleum ether/ethyl acetate (7:3).

### 3. Refinement

The H atom of the NH group was located in a difference map and later refined freely. The other H atoms were positioned with idealized geometry using a riding model with C—H = 0.93–0.96 Å. All H atoms were refined with isotropic displacement parameters (set to 1.2 times of the *U*_eq_ of the parent atom).

### Crystal data

**Table:**

C_7_H_6_F_3_NO_2_	prism
*M**_r_* = 193.13	*D*_x_ = 1.576 Mg m^−^^3^
Monoclinic, *C*2/*m*	Melting point: 405 K
Hall symbol: -C 2y	Mo *K*α radiation, λ = 0.71073 Å
*a* = 16.643 (2) Å	Cell parameters from 645 reflections
*b* = 7.1118 (10) Å	θ = 3.0–24.4°
*c* = 6.9618 (11) Å	µ = 0.16 mm^−^^1^
β = 98.903 (7)°	*T* = 293 K
*V* = 814.1 (2) Å^3^	Prism, colourless
*Z* = 4	0.24 × 0.22 × 0.20 mm
*F*(000) = 392	

### Data collection

**Table:**

Bruker APEXII CCD diffractometer	707 independent reflections
Radiation source: fine-focus sealed tube	645 reflections with *I* > 2σ(*I*)
Graphite monochromator	*R*_int_ = 0.076
Detector resolution: 1.08 pixels mm^-1^	θ_max_ = 24.4°, θ_min_ = 3.0°
ω scans	*h* = −19→19
Absorption correction: multi-scan (*SADABS*; Bruker, 2009)	*k* = −8→8
*T*_min_ = 0.963, *T*_max_ = 0.969	*l* = −3→7
3752 measured reflections	

### Refinement

**Table:**

Refinement on *F*^2^	Secondary atom site location: difference Fourier map
Least-squares matrix: full	Hydrogen site location: inferred from neighbouring sites
*R*[*F*^2^ > 2σ(*F*^2^)] = 0.065	H atoms treated by a mixture of independent and constrained refinement
*wR*(*F*^2^) = 0.180	*w* = 1/[σ^2^(*F*_o_^2^) + (0.1299*P*)^2^ + 0.3175*P*] where *P* = (*F*_o_^2^ + 2*F*_c_^2^)/3
*S* = 1.09	(Δ/σ)_max_ < 0.001
707 reflections	Δρ_max_ = 0.37 e Å^−^^3^
81 parameters	Δρ_min_ = −0.32 e Å^−^^3^
0 restraints	Extinction correction: *SHELXL*, Fc^*^=kFc[1+0.001xFc^2^λ^3^/sin(2θ)]^-1/4^
0 constraints	Extinction coefficient: 0.08 (2)
Primary atom site location: structure-invariant direct methods	

### Special details

**Table:**

Geometry. All e.s.d.'s (except the e.s.d. in the dihedral angle between two l.s. planes) are estimated using the full covariance matrix. The cell e.s.d.'s are taken into account individually in the estimation of e.s.d.'s in distances, angles and torsion angles; correlations between e.s.d.'s in cell parameters are only used when they are defined by crystal symmetry. An approximate (isotropic) treatment of cell e.s.d.'s is used for estimating e.s.d.'s involving l.s. planes.
Refinement. Refinement of *F*^2^ against ALL reflections. The weighted *R*-factor *wR* and goodness of fit *S* are based on *F*^2^, conventional *R*-factors *R* are based on *F*, with *F* set to zero for negative *F*^2^. The threshold expression of *F*^2^ > σ(*F*^2^) is used only for calculating *R*-factors(gt) *etc*. and is not relevant to the choice of reflections for refinement. *R*-factors based on *F*^2^ are statistically about twice as large as those based on *F*, and *R*- factors based on ALL data will be even larger.

### Fractional atomic coordinates and isotropic or equivalent isotropic displacement parameters (Å^2^)

**Table:**

	*x*	*y*	*z*	*U*_iso_*/*U*_eq_	Occ. (<1)
H	0.275 (3)	0.0000	−0.341 (8)	0.073 (13)*	
C1	0.0515 (2)	0.0000	0.2701 (6)	0.0765 (12)	
H1A	0.0456	0.1254	0.3171	0.115*	0.50
H1B	0.0701	−0.0816	0.3777	0.115*	0.50
H1C	0.0000	−0.0438	0.2042	0.115*	0.50
C2	0.18890 (17)	0.0000	0.2149 (4)	0.0436 (9)	
C3	0.24211 (16)	0.0000	0.0673 (4)	0.0391 (8)	
C4	0.21641 (19)	0.0000	−0.1280 (4)	0.0480 (8)	
H4	0.1625	0.0000	−0.1881	0.058*	
C5	0.3504 (2)	0.0000	−0.0863 (5)	0.0563 (10)	
H5	0.4034	0.0000	−0.1137	0.068*	
C6	0.32889 (16)	0.0000	0.0952 (4)	0.0443 (8)	
C7	0.38690 (18)	0.0000	0.2762 (5)	0.0544 (9)	
F1	0.46353 (14)	0.0000	0.2439 (4)	0.1048 (12)	
F2	0.38017 (10)	0.1475 (2)	0.3904 (2)	0.0853 (8)	
N	0.28189 (18)	0.0000	−0.2200 (5)	0.0587 (9)	
O1	0.20994 (15)	0.0000	0.3872 (3)	0.0718 (9)	
O2	0.11034 (12)	0.0000	0.1358 (3)	0.0586 (8)	

### Atomic displacement parameters (Å^2^)

**Table:**

	*U*^11^	*U*^22^	*U*^33^	*U*^12^	*U*^13^	*U*^23^
C1	0.057 (2)	0.116 (3)	0.058 (3)	0.000	0.0154 (17)	0.000
C2	0.0503 (16)	0.0517 (15)	0.026 (2)	0.000	−0.0038 (11)	0.000
C3	0.0478 (15)	0.0422 (13)	0.0235 (19)	0.000	−0.0066 (11)	0.000
C4	0.0574 (17)	0.0589 (16)	0.0237 (19)	0.000	−0.0065 (12)	0.000
C5	0.0570 (17)	0.0708 (19)	0.042 (2)	0.000	0.0092 (14)	0.000
C6	0.0491 (16)	0.0483 (14)	0.0331 (18)	0.000	−0.0007 (11)	0.000
C7	0.0481 (16)	0.0667 (17)	0.045 (2)	0.000	−0.0052 (13)	0.000
F1	0.0486 (13)	0.185 (3)	0.076 (2)	0.000	−0.0056 (11)	0.000
F2	0.0959 (13)	0.0903 (13)	0.0570 (14)	−0.0019 (7)	−0.0278 (8)	−0.0240 (7)
N	0.077 (2)	0.0781 (18)	0.020 (2)	0.000	0.0034 (13)	0.000
O1	0.0645 (15)	0.125 (2)	0.0230 (17)	0.000	−0.0013 (10)	0.000
O2	0.0470 (13)	0.0914 (16)	0.0352 (16)	0.000	−0.0006 (9)	0.000

### Geometric parameters (Å, º)

**Table:**

C1—O2	1.455 (4)	C4—H4	0.9300
C1—H1A	0.9600	C5—N	1.356 (5)
C1—H1B	0.9600	C5—C6	1.366 (5)
C1—H1C	0.9600	C5—H5	0.9300
C2—O1	1.196 (4)	C6—C7	1.464 (5)
C2—O2	1.338 (4)	C7—F1	1.329 (4)
C2—C3	1.457 (4)	C7—F2^i^	1.332 (3)
C3—C4	1.360 (4)	C7—F2	1.332 (3)
C3—C6	1.427 (4)	N—H	0.84 (5)
C4—N	1.347 (4)		
			
O2—C1—H1A	109.5	N—C5—H5	125.6
O2—C1—H1B	109.5	C6—C5—H5	125.6
H1A—C1—H1B	109.5	C5—C6—C3	106.2 (3)
O2—C1—H1C	109.5	C5—C6—C7	124.3 (3)
H1A—C1—H1C	109.5	C3—C6—C7	129.5 (3)
H1B—C1—H1C	109.5	F1—C7—F2^i^	105.81 (19)
O1—C2—O2	121.9 (3)	F1—C7—F2	105.81 (19)
O1—C2—C3	126.3 (3)	F2^i^—C7—F2	104.0 (3)
O2—C2—C3	111.8 (3)	F1—C7—C6	112.2 (3)
C4—C3—C6	106.9 (3)	F2^i^—C7—C6	114.12 (17)
C4—C3—C2	125.0 (3)	F2—C7—C6	114.12 (17)
C6—C3—C2	128.1 (3)	C4—N—C5	109.3 (3)
N—C4—C3	108.8 (3)	C4—N—H	119 (3)
N—C4—H4	125.6	C5—N—H	131 (3)
C3—C4—H4	125.6	C2—O2—C1	116.6 (3)
N—C5—C6	108.8 (3)		

Symmetry code: (i) *x*, −*y*, *z*.

### Hydrogen-bond geometry (Å, º)

**Table:**

*D*—H···*A*	*D*—H	H···*A*	*D*···*A*	*D*—H···*A*
N—H···O1^ii^	0.83 (6)	2.03 (5)	2.810 (4)	156
C5—H5···F1^iii^	0.93	2.52	3.442 (4)	171

Symmetry codes: (ii) *x*, *y*, *z*−1; (iii) −*x*+1, *y*, −*z*.
